# Meetings of the Bristol Medico-Chirurgical Society

**Published:** 1892-06

**Authors:** 


### Bristol Medico-Chirurgical Society.

*April* 13*th*, 1892.

Mr. F. R. Cross, President, in the Chair.

Dr. Michell Clarke showed a specimen, with microscopic sections, of a tumour of the base of the brain. It occurred in a man who had previously had syphilis; and, besides other symptoms, gave rise to bitemporal hemianopsia. Later he became blind and deaf. The growth was connected with the dura mater, was moderately firm, and microscopically was a so-called "endothelioma." He also showed a heart with several primary growths in the muscular substance, especially in walls of left ventricle and left auricle. There was no tumour elsewhere in body. These growths were oval and spindle-celled sarcoma.

Dr. Elliott read notes on a case of tumour of the pons Varolii, occurring in a girl aged nine. The symptoms were sickness, vertigo, frontal headache, internal squint, paresis of left side of body, and finally coma. The ventricles were found *post mortem* to be much distended, and a glioma was situated on the pons.

Mr. Ewens showed a female patient with long-standing scirrhus of left breast and subcutaneous nodules scattered over the upper part of the chest.

Dr. Leonard Lees described a case of intestinal obstruction, and showed a specimen. The patient, a healthy young man, was attacked with violent abdominal pain and nausea after an indigestible meal. The symptoms partially subsided, but there was no passage of motion or flatus. He became collapsed, and died fifty hours after onset of the pain. It was found that three and a half feet of the ileum just above the ileo-cæcal valve were strangulated by a band stretching from two points on the anterior abdominal wall near the right internal ring.

Dr. Lees also showed a specimen of gangrene of the vermiform appendix from a patient who was suddenly seized with sickness, diarrhœa, and severe abdominal pain. There was no localised tenderness. The symptoms improved, but on the fifth day of the illness there was sudden collapse and death. At the *post mortem* examination the appendix, with two perforations, was found lying in the pelvis, surrounded by signs of extensive peritonitis.

Dr. Roxburgh brought the following case before the Society: A lady, aged 37, was attacked with symptoms of gastro-duodenitis, obstinate constipation, and asthenia in the seventh month of her ninth pregnancy. The urea secreted was diminished; there was no albumin or sugar. She died comatose after giving birth to a child. The liver was found to be of a bright yellow colour, of the consistence of putty, and structureless, but enlarged. The increase in size appeared to be due to the presence of fat. Dr. Roxburgh considered the case to be one of acute yellow atrophy of the liver in an early stage.

---

*May 11th*, 1892.

Mr. F. R. Cross, President, in the Chair.

Dr. Markham Skerritt described a case of transposition of the viscera: the heart and spleen and apparently the stomach were on the right side, and the liver on the left.

Dr. Ewen Maclean showed a boy (brought before the Society also on November 11th) with morbus maculatus. There were three hemorrhages on the retina to be detected.

Dr. Nevill read notes of a case of puerperal pulmonary obstruction. The characteristic symptoms of livid face, gasping respirations, and quickened pulse came on forty-eight hours before the commencement of labour. Delivery was effected as speedily as possible. Phlegmasia dolens followed two days after. The woman recovered. Dr. Nevill mentioned various theories to account for the plugging of the pulmonary artery before the thrombosis in the legs.

Mr. Pickering described a case of nephro-lithotomy in a woman aged 25. Symptoms pointed to stone in the right kidney, and an exploratory opening was made in the loin and a quantity of pus evacuated, together with twenty-three calculi of various sizes. The patient made a good recovery, but still has a sinus discharging urine.

Mr. Greig Smith exhibited a round stone, coated with crystalline deposit, which he had removed by nephro-lithotomy.

Dr. Edgeworth showed specimens of: (1) Sarcoma of the ileo-cæcal valve, which gave rise during life to attacks of abdominal pain, &c.; (2) Strangulation of Meckel's diverticulum by the diverticulum cord. This caused symptoms of acute intestinal obstruction; (3) Partial intussusception of the vermiform appendix. He also showed a child with head-nodding and nystagmus.

Mr. Morton showed a child with symmetrical enlargement of several joints (knees, elbows, &c.). He considered the condition one of pulpy disease, but the diagnosis was uncertain.

Mr. Morton (for Dr. Barrett Roué) exhibited a specimen of fæcal fistula into the peritoneal cavity. There was no sign of tubercular disease, and the cause of the perforation was obscure.

G. Munro Smith, *Hon. Sec.*

